# 1,25-Dihydroxyvitamin D_3_ Protects against Immune-Mediated Killing of Neurons in Culture and in Experimental Autoimmune Encephalomyelitis

**DOI:** 10.1371/journal.pone.0144084

**Published:** 2015-12-17

**Authors:** Scott Sloka, Simon Zhornitsky, Claudia Silva, Luanne M. Metz, V. Wee Yong

**Affiliations:** Hotchkiss Brain Institute and the Department of Clinical Neurosciences, Cumming School of Medicine, University of Calgary, Calgary, Canada; Universite Lyon 1, FRANCE

## Abstract

Several studies have reported that low vitamin D levels are associated with an increased risk of developing multiple sclerosis (MS). As MS is an inflammatory disorder with degeneration of axons and neurons, we examined whether the biologically active form of vitamin D, 1,25-dihydroxyvitamin D_3_ (1,25D_3_), could protect against the T cell-mediated killing of human neurons in culture, and the axonal loss seen in mice with experimental autoimmune encephalomyelitis (EAE). Human neurons were exposed to activated human T lymphocytes and the loss of neurons was documented 24 hours later by counting the number of microtubule-associated protein-2 positive cells. Mice with EAE were harvested for counts of axonal profiles in the spinal cord. 1,25D_3_ was exposed to T cells in culture or administered to mice from peak EAE clinical severity when axonal loss was already evolving. Activated T lymphocytes killed human neurons prominently within 24 hours but toxicity was significantly attenuated when T cells were exposed to 1,25D_3_ prior to the co-culture. In EAE, 1,25D_3_ treatment initiated from peak clinical severity reduced the extent of clinical disability and mitigated the progressive loss of axons. The reduction of axonal and neuronal loss by 1,25D_3_ in the context of an inflammatory assault to the central nervous system is a potential contributor to the putative benefits of vitamin D in MS.

## Introduction

Multiple sclerosis (MS) is an inflammatory and neurodegenerative disorder with widespread demyelination and axonal loss within the central nervous system (CNS). The underlying etiology remains undefined although both environmental and genetic factors play a role; familial inheritance, cigarette smoking, viral infection and deficient ultraviolet light exposure may all contribute to the elevated risk of MS [1–4]. Regardless of the initiating etiology, there is over-activation of various immune subsets and they accumulate in the CNS to produce injury. For example, antigen- or non-antigen-reactive T cells that have been activated are potent mediators of neuronal death in culture and in animal models of MS [5–7].

Several studies have reported an inverse association of sunlight exposure, available UV radiation and MS prevalence–an effect hypothesized to be mediated by vitamin D [8–10]. More recently, a meta-analysis of 11 studies (1007 patients) showed that low vitamin D levels are associated with an increased risk of MS [11]. The exact mechanisms for putative benefit of vitamin D in MS still need to be elucidated. Interestingly, preclinical studies suggest that vitamin D possesses both immunomodulatory and non-immunomodulatory neuroprotective properties. For example, vitamin D increases the proliferation of regulatory T cells while reducing that of pro-inflammatory T cells, skews T cells towards production of the anti-inflammatory T helper (Th) 2 subset, and reduces the severity of experimental autoimmune encephalomyelitis (EAE), an inflammatory model of MS [12–13]. Additionally, vitamin D reduces ischemic cell death in primary cortical neurons [14], attenuates glutamate-induced excitotoxic cell death [15] and protects against neurotoxicity by rotenone in SH-SY5Y cells [16]. There is also evidence that vitamin D can enter the CNS [17–18]. A direct effect of vitamin D on the CNS is supported by studies showing that mice treated with vitamin D exhibit significantly reduced demyelination and increased remyelination in the cuprizone model, which is a non-inflammatory model of white matter damage [19–20].

In order to further elucidate the mechanisms associated with putative benefits of vitamin D for MS, we tested the hypothesis that its biologically active form, 1,25-dihydroxyvitamin D_3_ (1,25D_3_), attenuates the T cell killing of neurons, thereby reducing the manifestation of a hallmark neuropathology in MS. Moreover, we evaluated whether 1,25D_3_ could reduce the axonal degeneration that occurs in EAE. Our results suggest a heretofore unknown neuroprotective role for 1,25D_3_ in inflammatory neurodegeneration.

## Methods

### Preparation of human fetal neurons

Brain tissues from human fetuses of 10–18 weeks fetal age were obtained following therapeutic abortion. Written informed consent from the donor or the next of kin was obtained by the medical team for the use of the fetal samples in research. The laboratory team had no access to the medical records other than the approximate age of the fetuses. The use of the fetal samples was approved by the Conjoint Health Research Ethics Board at the University of Calgary. For the preparation of brain cell cultures, which we detailed previously [21], 5–15g of brain tissue diced into fragments of <1 mm with a pair of scalpels was incubated for 15min at 37°C with 0.25% trypsin and 200μg/ml DNase I in PBS (phosphate buffered solution). The suspension was then washed through a filter of 130-μm pore size, and the filtrate was centrifuged at 1200rpm for 10min. The cell pellet was resuspended in PBS and centrifuged. Following a final washing step in feeding medium (see below), the pellet was suspended in feeding medium and cells were plated into T-75 flasks coated with 10μg/ml polyornithine. Plating density was 50 million cells in 25ml of medium. Feeding medium was MEM-supplemented with 10% fetal bovine serum, 1% penicillin/streptomycin, 20μg/ml gentamicin, 0.1% dextrose, 1X non-essential amino acids, 10μM glutamine, and 1μM sodium pyruvate. All medium constituents were from Invitrogen Life Technologies.

To obtain neuron-enriched cultures, cells in the T-75 flasks were subjected, immediately upon seeding, to 3 cycles of 25μM cytosine arabinoside (Sigma-Aldrich, St. Louis, MO) to kill dividing astrocytes. Each cycle consisted of 2 days of treatment with cytosine arabinoside, followed by 3 days of non-cytosine arabinoside containing feeding medium. Neuron-enriched cultures, in excess of 80% purity, were then plated into wells of 96-well plates at a density of 100,000 cells per 100μl of medium per well. Another treatment with cytosine arabinoside ensued, and cells were used for cytotoxicity assays at least 2 days after removal of cytosine arabinoside-containing medium.

### Isolation of T Cells

Peripheral blood mononuclear cells (PBMCs) were isolated from the blood of healthy adult volunteers by Ficoll-Hypaque centrifugation. Written informed consent from the donor or the next of kin was obtained for the use of the PBMC samples in research. The PBMCs were washed once with PBS and suspended in serum-free AIM-V medium (Invitrogen Life Technologies, Burlington, Ontario, Canada). To activate T cells in the PBMC populations, 96 well round-bottomed plates were coated with 10 or 1000ng/mL of purified mouse anti-human CD3 (BD Pharmingen, Franklin Lakes, NJ) for a period of 3h. From previous experiments (data not shown), the coating at 1000ng/mL of anti-CD3 gives maximal activation of human T cells measured by proliferation assays. Since the *in vivo* environment in humans is unlikely to lead to the maximal activation of T cells, a submaximal level of activation with 10ng/ml anti-CD3 was also used in most experiments as both a comparison to maximal activation and to better reflect physiology. This submaximal level of activation may also permit the more sensitive measurement of experimental changes that affect T cell activation.

PBMCs were plated at a density of one million cells/mL of anti-CD3 coated 96 well plates (200 μl/well, 200,000 cells per well). An additional 10ng/ml of anti-CD28 (BD Pharmingen) was added as a suspension to all cultures, and cells were left for 3 days at 37°C in a 5% humidified CO_2_ incubator. Specified sister cultures were further exposed to either 0.1, 1 or 10nM of 1,25D_3_ (1,25-dihydroxyvitamin D_3_,purchased from BioMol, Plymouth Meeting, PA). In some experiments, certain PBMC preparations did not receive anti-CD3 or 1,25D_3_, and the floating cells collected 3 days thereafter were referred to as unactivated T cells.

Flow cytometry analyses of the floating cells collected after 3 days of the initiation of anti-CD3 treatment indicated that CD3+ T cells constituted approximately 90% of the total cell population (data not shown). Of the CD3 cells, 60% were CD4+ and 40% were CD8+. For the remaining cells, approximately 8% were CD56+ natural killer T lymphocytes, approximately 2% were CD19+ B lymphocytes, and less than 1% were CD14+ monocytes. There was no significant difference in the proportion of the various cell subsets between the unactivated, 10 and 1000ng/mL anti-CD3 activated PBMC populations (data not shown). Since the majority of cells were T cells, henceforth this culture population is referred to as T cells.

### Addition of cells to neurons

Since neurons are destroyed equally by either syngeneic T cells from the spleen of the brain donor, or by allogeneic T cells from PBMCs derived from the peripheral blood of adult human donors [5], the latter was used in the current study. T cells were either unactivated or activated with 1000ng/ml of anti-CD3 with 10 ng/ml of soluble anti-CD28, and left for 3 days at 37°C in a 5% humidified CO_2_ incubator. Additional sets of the anti-CD3 activated T cell populations were treated with either 0.1, 1 or 10nM of 1,25D_3_ from the time of exposure to anti-CD3. After 72h, T cells were collected, counted, and added to the neuron culture at a concentration of 100,000 cells per well (neurons to T cell ratio is 1:1) as follows: neurons alone; neurons with unactivated T cells; neurons with activated T cells; neurons with activated T cells exposed to three increasing concentrations of 1,25D_3_. Three different sets of co-cultures segregated by the nature of 1,25D_3_ treatment were created on the same plate: neurons with T cells pre-treated for 3 days with 1,25D_3_; neurons with T cells pre-treated with 1,25D_3_, and with further 1,25D_3_ added during co-culture (“pre- and post-treated”); and neurons with T cells where 1,25D_3_ was added only at the time of co-culture (“post-treated”). Cultures were fixed in 4% paraformaldehyde after 24h of co-culture for 15 minutes. Cultures were stained for microtubule associated protein 2 (MAP-2, Sigma, St. Louis, MO) to identify neurons, and with Hoescht dye to identify nuclei.

The quantitation for neuronal counts (cells positive for both MAP-2 and Hoescht dye) was performed using ImageXpress Micro Cellular Imaging & Analysis System (Molecular Devices, Sunnyvale, CA) [22]. The same four sites per well that were close to the center were selected for all wells, and results were pooled for each well. Four wells per treatment were used per experiment.

### Impact of 1,25D_3_ on growth factor production by neurons

Enriched human neurons after cytosine arabinoside treatment were plated in poly-l-lysine-coated wells of 6-well plates at a density of 2.5 million cells/well. A day after, cells were treated with vehicle or 1,25D_3_ for 24h, and RNA was then extracted using TRIzol solution (Life Technologies, Carlsbad, CA). Real time polymerase chain reaction (PCR) for growth factors was performed as previously described [23]. All primers were obtained from SABiosciences, a QIAGEN company (VEGF, # PPH00251C, BDNF,# PPH00569E, IGF-1, #PPH00167B, NT-3#, #PPH00687A).

### Disease induction and EAE analysis

Animal studies were performed according to ethical policies and approved by the Animal Care Committee at the University of Calgary, in accordance with research guidelines from the Canadian Council for Animal Care. All efforts were made to minimize suffering, such as by the use of anesthetic during the immunization procedure or the oral gavage of fluids for hydration in disabled mice, and we sought also to use the minimal number of mice required. We planned to sacrifice animals for humane reasons if they were paralysed but this was not needed in our experiments.

EAE was induced in female C57BL/6 mice (Jackson Laboratories, Bar Habour, Maine), aged 8–9 weeks, by injecting subcutaneously (s.c.) 50μg of myelin oligodendrocyte glycoprotein peptide (MOG)_35–55_ in Complete Freund's Adjuvant (CFA) (Fisher, Michigan USA) on day 0 [24–25]. Intraperitoneal (i.p.) pertussis toxin (0.1μg/200μl, List Biological labs, Hornby, ON) was administered on days 0 and 2. We used a total of 36 mice for our experiments. All 36 mice were immunized on the same day (day 0) with MOG, and 4 mice were sacrificed at day 18, representing the time period of expected peak clinical severity, for baseline histology. The remaining 32 mice were equally and randomly divided into 1,25D_3_ or vehicle treatment groups at day 18, when treatment was initiated. 1,25D_3_ (100ng per mouse) was given every other day intraperitoneally in 50μl in 100% DMSO, while 50μl of 100% DMSO was used as the vehicle control. Treatment ensued until the termination of the experiment at day 56.

Animals were assessed daily using a 15-point disease score scale [24,25] replacing the more commonly used 5-point scale since the 15-point scale differentiates individual limb disability, rather than grouping both fore- or hind-limbs together. This allows for a more sensitive characterization of disease progression. The 15-point scale is the sum of the disease state for the tail (scored from 0–2) and all 4 limbs (each limb is scored from 0–3).

### Histology of tissue from control and EAE mice

At day 56, all EAE animals were killed via an overdose of ketamine/xylazine. Spinal cords were removed, placed in formalin, and thoracic (1.0cm) blocks were then embedded in paraffin. Cords were cut on a microtome at 6μm thickness and mounted on glass slides. For each mouse, the thoracic cord was cut longitudinally through the entire dorsal–ventral axes, to obtain a total of six sequential series of sections; adjacent sections for each series are spaced 50μm apart. One series was processed for Bielchowsky silver stain for axons [22]. An algorithm was then developed in Matlab to measure the axons. Briefly, the images were thresholded using a local adaptive filter to provide a measure of the stained axons. A transection perpendicular to the central canal was drawn through the lateral white matter only and the number of axons along the transection was counted for each image. Three areas (rostral, middle and caudal) of the lateral column were imaged using light microscopy so that three anatomically separate sections per animal were analyzed and averaged. This method has been described in a previous publication [22].

For each mouse, two axial sections 2mm thick were also taken at the lumbar level of L1, fixed in glutaraldehyde, and then embedded in plastic. These sections were processed and stained for toluidine blue to highlight the myelin in the axial plane. Images were captured using an Olympus BH-2 microscope (Olympus, Port Moody, Canada) and QCapture Pro software (version 5.1.1.14; Media Cybernetics Inc., Bethesda, MD).

To count the number of myelinated axons in the toluidine blue-stained sections, an algorithm was developed in Matlab to identify the number of axons within a given area by processing the images. Briefly, the algorithm uses local adaptive thresholding to identify unstained axonal regions that are encircled by stained myelin. These regions are filtered first by shape (highly concave regions are filtered out to remove non-axonal elements such as blood vessels), and then by size (to exclude any further non-axonal elements). Regions were then counted using standard Matlab routines, after the axons within myelin were false-colored red. The dorsal column of each section was oriented to maximize the number of axons imaged. The dorsal columns were selected such that the main portion of the columns highlighted was cut off at the inflection of the columns to the lateral tails, in order to standardize the selected region.

### Statistical analysis

Statistical analysis was performed using SPSS Statistics v.22.0 (IBM Corporation, Armonk, NY, USA, 2013) and Matlab version 7.7 (The Mathworks, Natick, MA, USA). Statistical differences for cells in culture and toluidine blue EAE axonal counts were addressed using ANOVA with Bonferroni correction for multiple comparisons. Statistical differences for Bielchowsky silver stain axonal counts/area in EAE were evaluated using a *t*-test for independent samples. Statistical analysis for the EAE clinical scores was conducted using the Mann-Whitney Wilcoxon test. An alpha of <0.05 was selected for statistical significance.

## Results

### 1,25D_3_ attenuates T cell killing of neurons

**Fig 1:**
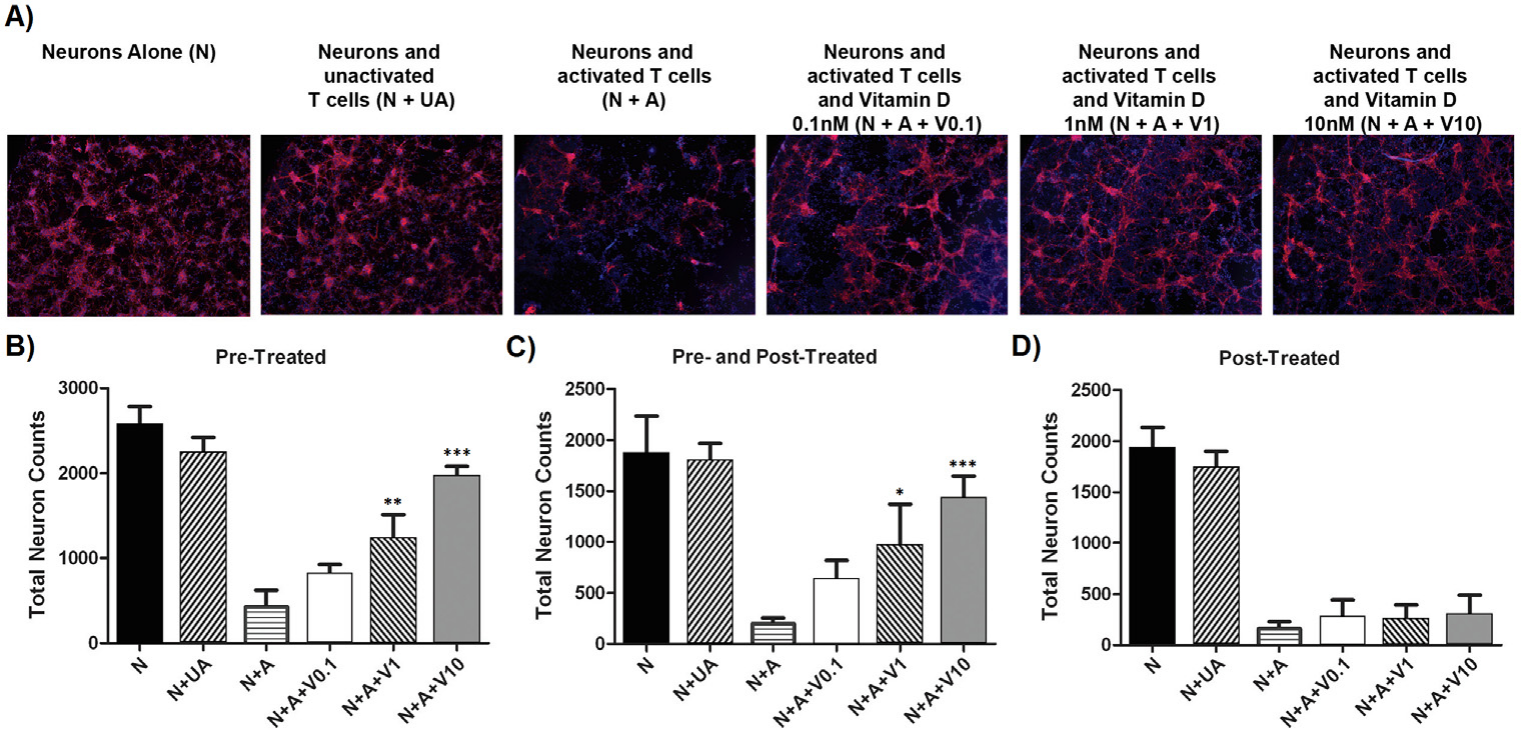
1,25D_3_ protects against T cell killing of neurons in culture. **(A,B)** Neurons were identified by immunofluorescence for MAP-2 (red). While neurons were not harmed after 24h by unactivated (UA) T cells, exposure to anti-CD3 activated T cells resulted in the loss of over 80% of neurons by 24h (N+A panel). Activated T cells exposed to increasing concentrations of 1,25D_3_ (abbreviated “V”) for 3 days prior to their co-culture with neurons were reduced in their capacity to kill neurons. **(C)** The protection by 1,25D_3_ against the T cell killing of neurons is also seen in the “pre- and post-treated” T cell group, but the post-treatment of T cells with 1,25D_3_ (i.e. at the time of T cell-neuron co-culture) lacks protective effect. Note that equal number (100,000) of control or 1,25D_3_-exposed T cells were added to neurons for their 24h of co-culture. Asterisks denote significance of 1,25D_3_ treatment, relative to activated T cells alone. Values are mean ± SD of quadruplicate analyses, and the results of the entire experiment were reproduced in another set; that 1,25D_3_-pretreatment of activated T cells alleviates their toxicity to neurons was observed in 3 experiments. * = p<0.05; ** = p<0.01; *** = p<0.001.

We examined whether 1,25D_3_ could protect against T cell killing of neurons *in vitro*. Fig 1a shows that while neurons were not harmed after 24h by unactivated T cells, exposure to anti-CD3 activated T cells resulted in the loss of over 80% of neurons. Significantly, activated T cells pre-treated with 1,25D_3_ for 3 days were attenuated in their capacity to kill neurons, and this occurred in a concentration-dependent manner (Fig 1b). Here, there was a significant difference between groups effect overall (p<0.0001). Post-hoc analysis revealed that the cultures treated with activated T cells contained significantly less neurons compared to when 1nM (p = 0.006) and 10nM (p<0.0001) of 1,25D_3_ was added to activated T cells prior to their exposure to neurons; however, the 0.1nM 1,25D_3_ was not protective (p>0.05).

The protection by 1,25D_3_ against the T cell killing of neurons is also seen in the “pre- and post-treated” T cell group (when 1,25D_3_ was exposed to T cells prior and during their co-culture with neurons) (Fig 1c). Here, there was a significant difference between groups effect overall (p<0.0001). Post-hoc analysis revealed that the neuronal pools treated with activated T-cells contained significantly less neurons compared to when 1nM (p = 0.014) and 10nM (p<0.0001) of 1,25D_3_ was added to them; however, the 0.1nM was not protective (p>0.05).

In order to determine whether the protection by 1,25D_3_ required its pre-treatment of activated T cells, a post-treated group was also created whereby activated T cells were added to neurons and then treated with varying concentrations of 1,25D_3_. Fig 1d shows that the post-treatment of T cells with 1,25D_3_ lacks the significant protective effect that pre-treated T cells demonstrate. Post-hoc analysis revealed that the neuronal pools treated with activated T cells did not contain significantly less neurons compared to when 0.1nM, 1nM, and 10nM (all p>0.05) of 1,25D_3_ was added to them.

### 1,25D_3_ produces an increase in neuronal growth factors

**Fig 2:**
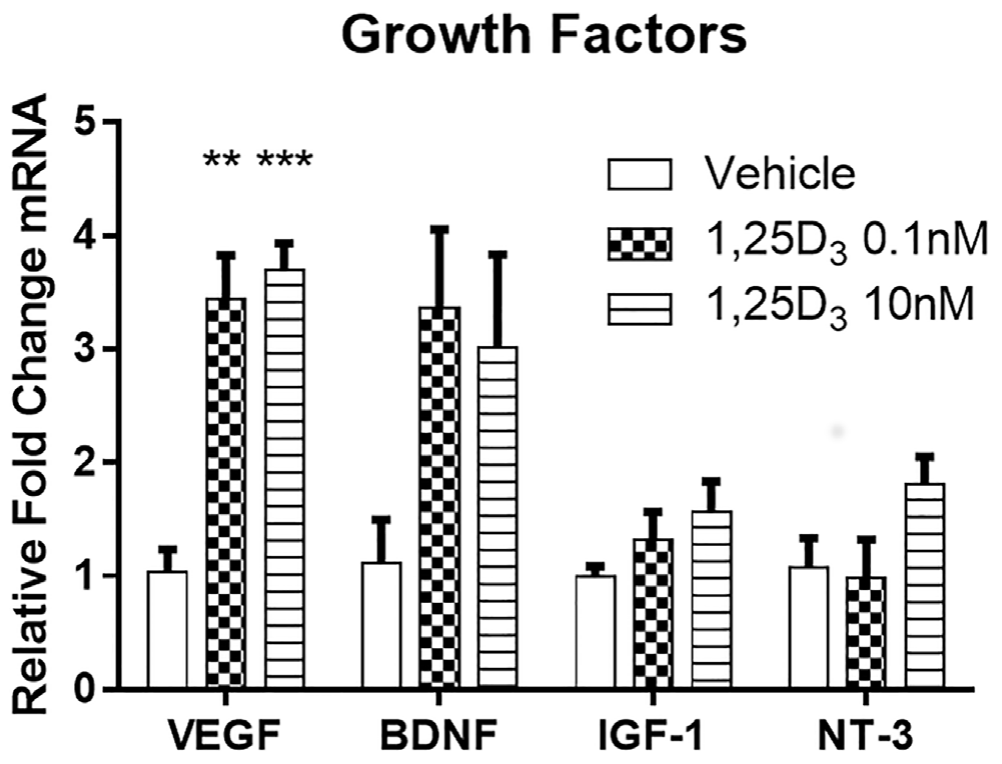
1,25D_3_ alters levels of growth factors in neurons. VEGF transcript levels were elevated by 1,25D_3_, relative to vehicle; there was a trend towards an increase of BDNF transcripts by 1,25D_3_ in neurons, but this was not statistically significant. Values are mean ± SD of triplicate analyses. **p<0.01, ***p<0.001 relative to vehicle.

To obtain further insights how 1,25D_3_ could be protective, we addressed whether it could directly impact human neurons. Following 24 hours of exposure to 1,25D_3_, neurons were subjected to PCR analyses of growth factors. The relative expression of mRNA for four different growth factors is shown in Fig 2. There was a between-groups effect (p = 0.001) and post-hoc analysis showed that 0.1nM (p = 0.003) and 10nM (p = 0.002) of 1,25D_3_ significantly increased VEGF levels, relative to vehicle. However, there were no significant between-group differences in levels of BDNF, IGF-1 or NT-3 when compared to vehicle (all p>0.05), although there was a trend of a 3-fold increase of BDNF with 1,25D_3_ treatment.

### Treatment of EAE mice at peak clinical disease with 1,25D_3_ produces sustained improvement in clinical score and sparing of axons

**Fig 3:**
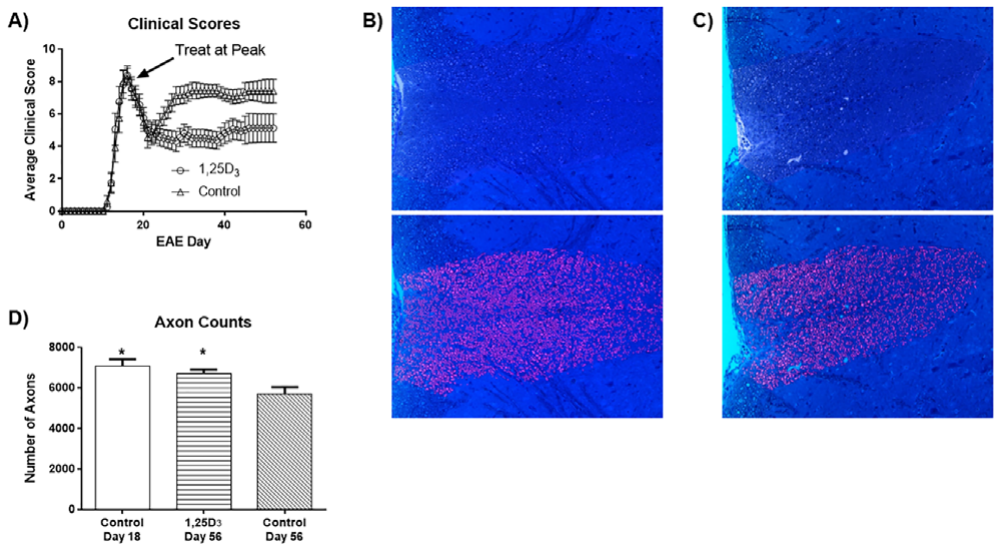
Treatment of EAE mice at peak disease demonstrates sustained improvement in clinical score and sparing of axons. Thirty-six mice were immunized with MOG, and 4 mice were sacrificed at day 18 for baseline histology. The remaining 32 mice were equally divided into 1,25D_3_ or vehicle treatment group until the end of the experiment where 8 mice each were randomly chosen for histological analyses. **(A)** 1,25D_3_ treatment initiated from peak clinical severity (day 18) caused a significant score separation from day 26 (p<0.05, Mann-Whitney Wilcoxon) that persisted for the remainder of the 56 days. **(B,C)** A representative image of toluidine blue-stained section (top) that was then processed to highlight axons (false-colored red) within myelin rings (see Methods for how the imaging analyses were performed). Panel B is from a 1,25D_3_-treated mouse, while panel C is from a vehicle-EAE animal. **(D)** 1,25D_3_ treatment of EAE mice leads to significant sparing of axons. Indeed, while control (vehicle) mice at day 56 displayed fewer axons than mice at day 18, indicating progressive axonal loss in the ensuing interval, this was prevented by 1,25D_3_. Values are mean ± SD. Asterisks denote significance of 1,25D_3_-treated EAE mice and axonal counts at day 18 (peak disease), relative to control day 56 EAE mice. * = p<0.05 compared to control day 56.

In a previous report, we noted that the treatment of mice with 1,25D_3_ from the day of MOG immunization (i.e. preventative treatment) prevented the appearance of clinical and histological signs of EAE [13]; we did not evaluate the impact of 1,25D_3_ treatment on the subsequent neuropathology of mice killed at day 20 of EAE in that study. Thus, to determine whether 1,25D_3_ could reduce the neurodegenerative process after axonal loss has been triggered, we examined the effect of 1,25D_3_ when initiated in mice demonstrating peak clinical disability (day 18 after MOG), and with continued treatment for every 2 days until harvest (day 56). Fig 3a shows that at 18 days after MOG-immunization, mice were at an average clinical score of 8 in the 15-point scale, which represents involvement of the tail and all 4 limbs. Following a slight recovery in clinical score after peak clinical severity, control mice (i.e., treatment with vehicle) hovered between scores 6 (impaired tail and paresis of both hindlimbs, with no forelimb involvement) to 8. 1,25D_3_ treatment improved EAE disability scores, which averaged 4 on the 15-point scale (impaired tail and minimal disability in both hindlimbs) until the experiment was terminated (day 56).

Tissues were processed for axonal counts in 2 ways. In the first, Epon-embedded sections were subjected to counts of axons enveloped within toluidine blue-stained myelin (Fig 3b and 3c). Baseline axonal counts of mice sacrificed at day 18, when 1,25D_3_ treatment was initiated, was first conducted. At this point, axonal loss in the white matter of the spinal cord was about 20% compared to naïve non-EAE mice [7]. A further attrition of axons was found when non-1,25D_3_ exposed mice were harvested at day 56 (Fig 3d, control mice). In contrast, 1,25D_3_-treated mice at the day 56 harvest had comparable axon counts as the day 18 control, suggesting that 1,25D_3_ had prevented axonal loss. There was a significant between-group effect overall (p = 0.013). Post-hoc analysis showed that axonal counts were significantly higher at day 18, compared with control day 56 mice (p = 0.025), but not compared with 1,25D_3_-treated mice (p>0.05). Moreover, mice treated with 1,25D_3_ until day 56 demonstrated significant preservation of axons, relative to control day 56 mice (p = 0.048).

**Fig 4:**
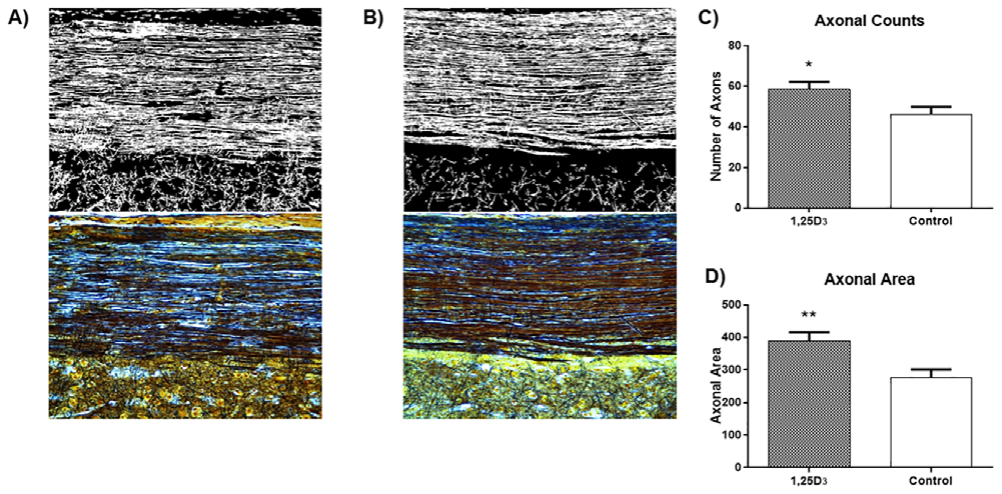
Treatment of EAE mice with 1,25D_3_ every other day from peak clinical disease reduces axonal loss at day 56. Thoracic spinal cord sections were subjected to Bielchowsky silver stains for axons. A section from each mouse landmarked by the central canal was digitalized (top panels, **A, B**) from the original view (bottom panels). Within Matlab, the images were thresholded using a local adaptive filter to provide a measure of the stained axons, a transection perpendicular to the central canal was drawn through the lateral white matter, and the number of axons along the transection was counted for each image, as used elsewhere [22]. Panel A is from a vehicle-EAE mouse while panel B is from a 1,25D_3_-treated animal. From the digitalized images (n of 8 mice per group, average of 3 images/mouse), the axonal counts **(C)** across the lateral column, as well as total axonal transectional diameter **(D)**, were obtained. Values are mean ± SD. Asterisks denote significance of axonal counts/area of 1,25D_3_-treated mice, relative to control mice. * = p<0.05; ** = p<0.01.

One criticism with the above method of counting is that active demyelination may obscure axons and therefore decrease the number of axons counted. We addressed this by directly labeling axons using Bielchowsky silver stains (Fig 4a and 4b). Fig 4 demonstrates that for mice sacrificed at day 56, with 1,25D_3_ or vehicle administered every other day from day 18, there was a relative preservation of axons in animals treated with 1,25D_3_. Specifically, 1,25D_3_-treated mice evidenced significantly higher axon counts (p = 0.026) and axonal area (p = 0.006), compared with control mice.

## Discussion

It has become clear that significant axonal injury and neuronal loss are major characteristics of the neuropathology of MS [26]. Indeed, these axonal and neuronal attrition are thought to drive the progression of disability in MS, and strategies to protect axons and neurons are sought as therapeutic approaches in the disease. Many factors contribute to axonal injury and neuronal demise in MS, including free radical-mediated toxicity, glutamate excitotoxicity, and T cell-mediated mechanisms [27]. We have reported that human T cells activated with anti-CD3 kill neurons during co-culture through contact-dependent mechanisms [5]. We therefore tested the hypothesis that one mechanism accounting for the benefits of vitamin D in MS is that it attenuates the T cell killing of neurons, thereby reducing the manifestation of a hallmark neuropathology in MS. In our co-cultures, we find that 1,25D_3_ significantly abrogates the T cell killing of neurons. This effect is shown in T cells that have been previously cultured with 1,25D_3_ and not with the addition of 1,25D_3_ at the time of co-culture (our post-treatment data).

Although vitamin D may be beneficial for MS due to suppression of the adaptive immune system, non-immune mechanisms may play a role as well. We therefore examined the effects of 1,25D_3_ on secretion of growth factors. We found that culturing neurons with 1,25D_3_ resulted in a significant increase of VEGF level. VEGF stimulates vasculogenesis which is necessary for repair, but also acts as a pro-inflammatory factor in the early phases of EAE and MS. However, in the late phases of disease, VEGF increases resistance of neurons to injury and regulates proliferation, migration, and differentiation of neural progenitors including oligodendrocyte precursor cells to demyelinated lesions [28].

BDNF and NT-3 are members of the neurotrophin family of growth factors, which are proteins that promote neuronal survival, development, and function and they may be stimulated by vitamin D [29]. Indeed, 1,25D_3_ significantly increased proliferation of neural stem cells, enhanced their differentiation into neurons and oligodendrocytes, and stimulated expression of growth factors, including BDNF and NT-3 [30]. Moreover, direct administration of BDNF using transformed bone marrow-derived stem cells reduced inflammatory infiltrating cells and attenuated apoptosis in the brain and spinal cord of EAE mice [31]. However, we did not find evidence of a significant elevation of BDNF or NT-3, and of IGF-1, in neuronal cultures exposed to 1,25D_3_.

In order to complement our *in vitro* experiments, we examined the effects of 1,25D_3_ in EAE. We found that treatment at peak disease elicited a significant score separation from day 26 that persisted for the remainder of the 56 days. Aggregate axonal analyses revealed that mice treated with 1,25D_3_ exhibited significant preservation of axons, relative to control mice. Indeed, the degeneration of axons in EAE from day 18 was countered when histological analyses were performed at day 56.

The beneficial effect of 1,25D_3_ in EAE has been well-established in previous studies. For instance, Lemire and Archer [32] showed that administration of 1,25D_3_ starting three days before immunization significantly prevented the development of EAE. Similarly, Cantorna et al. [33] revealed that 1,25D_3_ prevented the progression of EAE when administered at the appearance of the first disability symptoms and withdrawal of 1,25D_3_ resulted in a resumption of the progression of EAE. More recently, we showed that 1,25D_3_ consistently generated human and murine Th2 cells in culture and prevented EAE manifestation when administered from the day of immunization of mice with MOG [13]. Altogether, these data suggest that treatment with 1,25D_3_ before EAE induction or from peak disease is effective at reducing disease severity and this effect may be mediated at least in part through the attenuation of T cell killing of neurons, as shown in our *in vitro* experiments.

In conclusion, we provide novel data of the spectrum of activity of 1,25D_3_, particularly its capacity to abrogate the T cell-mediated killing of neurons and its amelioration of axonal loss in EAE. Our results provide further impetus to support the adequate supplementation with vitamin D for well-being in MS.
